# Physicochemical characteristics of lakes along the southern Baltic Sea coast

**DOI:** 10.1038/s41597-024-03195-2

**Published:** 2024-04-06

**Authors:** Krystian Obolewski, Mikołaj Matela, Katarzyna Glińska-Lewczuk, Aleksander Astel, Martyna Bąkowska-Hopcia

**Affiliations:** 1 Department of Hydrobiology, Kazimierz Wielki University, Bydgoszcz, Poland; 2 Department of Water Resources and Climatology, University of Warmia and Mazury, Olsztyn, Poland; 3 Environmental Chemistry Research Unit, Pomeranian University in Słupsk, Słupsk, Poland

**Keywords:** Environmental monitoring, Limnology

## Abstract

We present a unique data set of selected physicochemical parameters characterizing the environment of the Baltic coastal lakes within Polish borders. The peculiarity of the system derives principally from the interaction of the lakes with a sea of relatively low salinity. In contrast to our best understanding of the state of biological knowledge, the abiotic parameters of coastal lakes along the southern Baltic Sea have never previously been so comprehensively supplemented. The database consists of physicochemical properties of 13 coastal lakes based on the analytical assessment of 419 water samples collected seasonally between 2014 and 2019. Water properties were analyzed according to the connection of the lakes to the Baltic Sea using a total of 23 predictors. The lakes were classified as closed, intermittently connected, or open. Based on the physicochemical data, a relationship between the hydrological connection between the lakes and the sea was determined. The data collected could be used to monitor ongoing global climate change at the biosphere level.

## Background & Summary

The coastal zone of the southern Baltic appears to be highly diversed in terms of the hydrographic features. The most noteworthy of these features are the zone’s coastal lakes, which are unique among the European Union’s lakes and protected under the Natura 2000 program^1^.

The Baltic Sea is unique. With an area of around 420,000 km^2^, it is the largest brackish water area in the world. Coastal abrasion is not observed in places where there are lakes on the southern coast of the Baltic Sea. These lakes are usually separated from the sea by strips of land (beaches, dunes) of varying width (up to 1,000 m) and only slight differences in height (up to 100 m). However, there are no cliffs or geological formations that could be damaged by the action of waves. Compared to other coastal areas of this sea, along the southern coast there are numerous coastal lakes, lagoons, and bays (CIEM 2018). There is also a complex river system, including the mouths of such major rivers as the Vistula, the Oder, and several coastal rivers (including the Słupia, Łupawa, and Wieprza), as well as numerous smaller watercourses and canals^2^. Coastal water bodies include large brackish lakes such as Łebsko, Resko, and Gardno and relatively small ones such as Kopań or Bukowo, as well as many freshwater water bodies such as Sarbsko, Wicko Przymorskie, and Dołgie Wielkie^2^. Many European countries, including those in the Baltic Sea catchment area, face the challenge of reducing pollution loads from anthropogenic sources and the need to meet the requirements of the *Water Framework Directive* (2000/60/EC) and the *Wastewater Directive* (91/271/EEC) to achieve a good ecological status of waters^3^. These measures aim, among other things, to expand and protect the Natura 2000 network areas, of which coastal lagoons and lakes (priority habitat 1150) of high natural value constitute priority habitats.

Aquatic ecosystems along the coast are the final component of river basins and are characterized by varying degrees of contact with the sea^4,5^. Coastal lakes are usually classified into one of the following three main types of ecosystems based on the degree of hydrological connection: open lagoons (with free connection to the sea), sporadically open/closed lagoons (only periodically fully open) and closed coastal lakes (without permanent connection to the sea). Coastal ecosystems can also be subdivided according to the salinity of the water: freshwater (limnetic), intermittently saline (limnetic/haline) and saline (haline). The Baltic coastal lakes can be divided into three main types: (1) open: with permanent connection to the sea through channels/river mouths; (2) intermittently connected: with periodic inflow of seawater thanks to the unblocking of buried channels; and (3) closed: permanently isolated; as well as several subtypes^5^. This hydrological connectivity leads to a variable exchange of substances between the individual ecosystem types, which influences their ecological status depending on the predominance of the marine or terrestrial factor^6–8^. The evaluation of the functioning of microhabitats shaped by the mixing of fresh and brackish water must be based on deterministic chaos theory. In lakes connected to the sea, the phenomenon of “windows of opportunity” created by seawater intrusion is frequently observed. Although it is obvious that significant differences will exist between oligohaline and limnetic ecosystems (alternative stable states), a large uncertainty was caused by the fact that coastal lakes are a transitional form between the two. It turned out that even small and short-term fluctuations in water salinity prevent them from reaching an ecosystem stability. Instead, so-called adaptive cycles can be observed in them. It has been shown that there are different transitional states (phases) in coastal lakes: 1) oligohaline-limnetic towards an increase in salinity and 2) limnetic-oligohaline towards water desalination, as well as stable states: oligohaline and limnetic. The authors first try to determine the mechanisms of coastal lakes based on pelagic processes^1^. Nevertheless, the constant salinity of the southern Baltic Sea (7-8 PSU) leads to the presence of stenohaline organisms, while the salinity in the lakes is generally lower and fluctuates, favoring euryhaline organisms. Therefore, the periodic increase in seawater salinity creates a “window of opportunity” effect for the immigration of marine organisms into the lakes^8^. Among others, there are: the phenomenon of backwash and brackish water intrusion, which causes a periodic change in water quality; the phenomenon of wind support, which hinders or even prevents the outflow of land water into the sea; and the phenomenon of water accumulation on the southern coasts of the Baltic Sea, which is particularly clear during autumn and winter storms. The above phenomena in turn result from the lakes’ location at the interface between land and sea, i.e. two areas that differ in their physical and chemical properties. As a result, all hydrometeorological phenomena here have a more dynamic and robust course and a strong impact on the functioning of ecosystems. Consequently, the coastal zone is a much more difficult region for conducting hydrological studies.

A sharp deceleration in a watercourse’s inflow into a coastal water body accelerates the mineralization of organic matter and causes the deposition of allochthonous and autochthonous matter in the bottom sediments^9^. For this reason, waters flowing into the sea through coastal lakes may be less loaded with nutrients and organic matter than they would otherwise be^10,11^. Thus, coastal lakes act as sedimentation basins by reducing the pollutant flux from the catchment^12–14^. However, over time, they become overloaded with nutrients, leading to die-offs^7,15,16^. Such declines in biological components further reduce the buffering capacity of the lakes and poses a measurable threat to the ecological status of their ecosystems^9,17^. The decline in biocenotic components, especially at the producer level, limits the coastal lakes’ ability to reduce the amount of nutrients flowing into the sea from the land depressions in which they are located. These lakes act as sedimentation basins (buffers) that protect the coastal marine areas from degradation. The increase in biocenotic components in the lakes, especially at the producer level, contributes to less nutrient runoff from the coastal lake catchment areas into the sea. Along with the salinity gradient, the nature of the studied water bodies changes; there is significant variability in abiotic parameters relative to this salinity gradient. Such an occurrence is usually implied by winds from the N, NNE or, less frequently, NE direction. This effect of wind direction is due to the orientation of the channels connecting them to the sea relative to the shoreline (an angle of ~90° prevails). Thus, intrusion occurs at the following frequencies: in the brackish-water lakes, constantly throughout the year in Ptasi Raj and for 276 days in Lebsko; in the transitional lakes, for 165 days in Gardno but for only 102 days in Kopan. As a rule, lower trophic predictors and higher values of ions associated with seawater intrusion are observed in brackish lakes. In freshwater lakes, however, the situation is reversed. The values are most varied in transitional lakes, where a freshwater phase interrupts the period of seawater intrusion. Managing the condition of these lakes therefore requires changes not only in the lakes but mainly in their catchment areas, in line with the concept of Ecohydrological Nature-based Solutions^18^. Therefore, one possible conservation measure is to modify and shape the land-use patterns of individual catchments to reduce pollutant loads flowing into coastal water bodies^19^. The effectiveness of such measures must be continuously monitored by comparing the concentrations of the most important aquatic environmental parameters on a medium- to long-term basis.

Coastal lakes and lagoons, characterized by their unique biotopes, have attracted intensive scientific study for many years due to their significant differences in physical and chemical factors^8,20,21^. This variety is mirrored in their relatively high species diversity. However, being zones where land and sea interact, they face escalating threats of degradation from climate change, including alterations in sea levels and sporadic marine intrusions^22–24^. To counteract this, there is a pressing need for comprehensive spatio-temporal data on water quality within these ecosystems to establish a dependable and integrated database^25^. Most research on the ecosystems of the lakes on the southern Baltic coast since the 20^th^ century has focused on the hydrology and hydrochemistry of the three largest lakes, Łebsko, Gardno, and Jamno^15,26–28^. In addition, several multidisciplinary studies have examined Lake Gardno^29^ and the lakes Gardno, Dołgie Małe, Dołgie Wielkie, and Łebsko^30^. However, it was only in the 2010s that studies began to include most Polish Baltic coastal lakes in terms of their hydrology^2^ and to carry out complex analyses of hydroecological conditions^1^. The state monitoring services do not carry out continuous monitoring of the indicated sites and, in the case of data for Lake Mikoszewskie, the current article consitutes the first scientific report. This causes many problems in tracking the transformations they undergo and in preparing active conservation programs that consider their evolutionary specifity. The database built as part of this research can be used to answer many questions about the condition of coastal lakes at different scales. It can be applied to investigate the influence of trophic levels on the ecologies of lakes on a salinity gradient^6^. We hope that our database will broaden the knowledge on the condition of this type of ecosystem at a global scale. The database is a unique compilation of data on many parameters of the coastal lakes along the southern Baltic Sea. Never before has the state of knowledge on this aspect been supplemented so comprehensively. Thus, the main idea of this study is to (i) provide information on the state of the biotope of coastal lake ecosystems; (ii) provide implications for comparative analyses of other ecosystems of this type in the world; (iii) establish a “0” state that will allow us to track changes through the evolutionary-ecosystem paradigm of ecosystem susceptibility to climate change.

## Methods

**Fig. 1:**
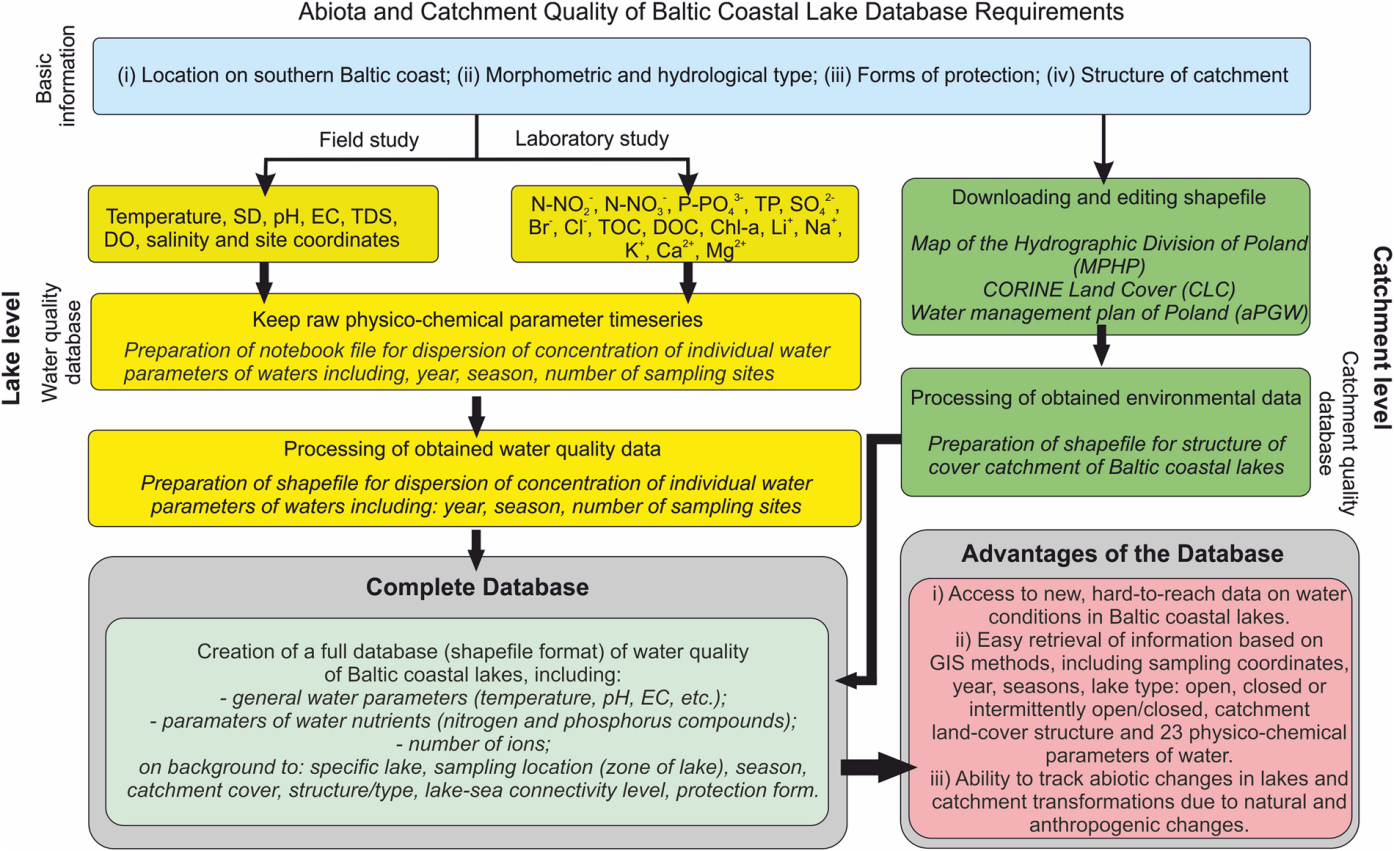
Workflow chart of steps used to create database. Describes the major decisions and activities involved in creating the database of physicochemical parameters for Baltic coastal lakes.

The organizational workflow chart is presented in Fig. 1, which includes the requirements for data-quality measures before data were merged to create a database of abiotic factors characterizing the ecological condition of biotopes.

### Sample acquisition

**Fig. 2:**
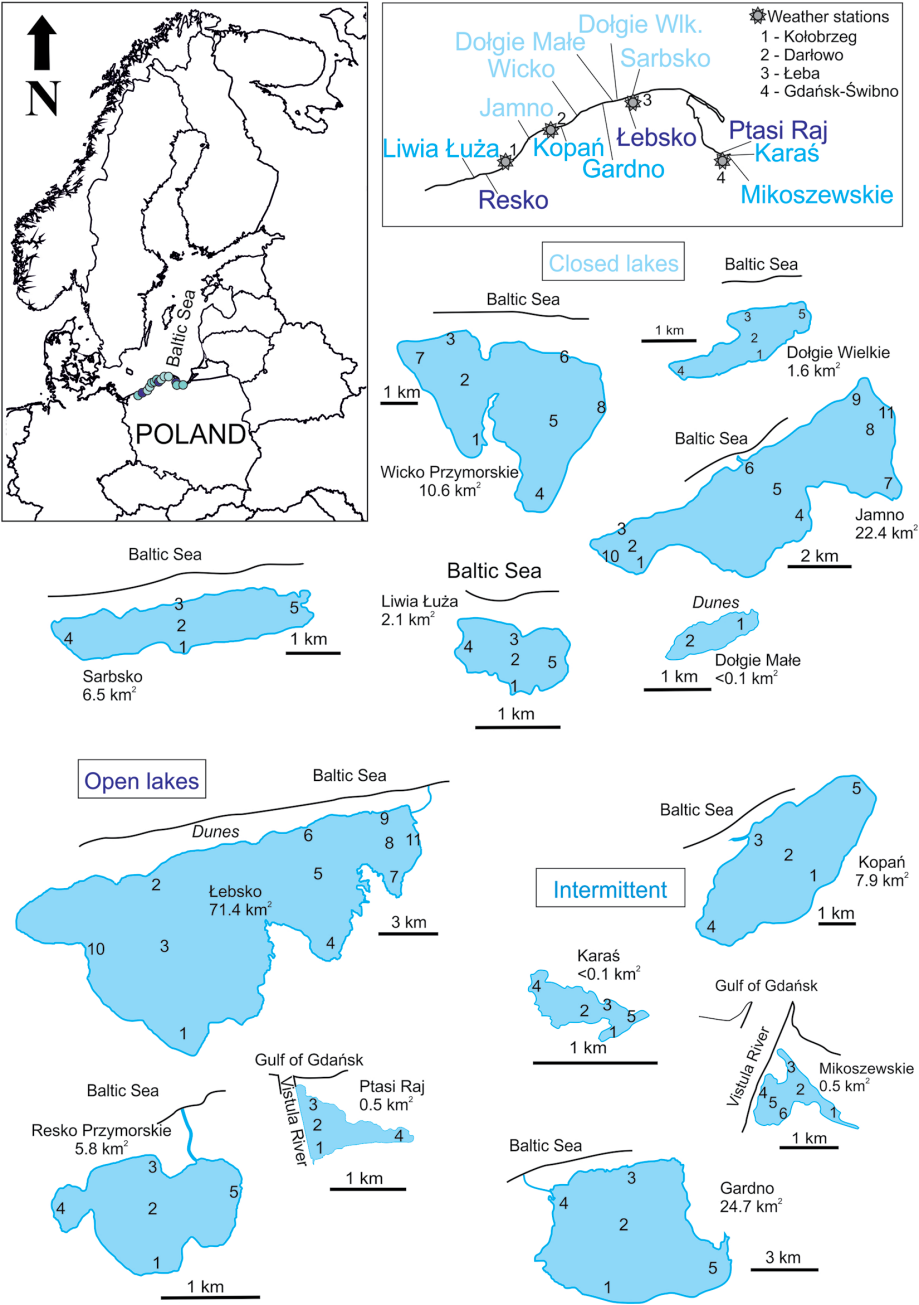
The studied Baltic coastal lakes, showing shape of individual lakes, location relative to coast, distribution of sampling points, geographical names and surface areas, and location in Poland.

Lake water samples were collected from 13 coastal lakes of the southern Baltic Sea coast (Liwia Łuża, Resko, Jamno, Kopań, Wicko, Gardno, Dołgie Małe, Dołgie Wielkie, Łebsko, Sarbsko, Ptasi Raj, Karaś, and Mikoszewskie). The lakes are highly polymictic, with average depths ranging from 0.7 m (Dołgie Małe Lake) to 2.7 m (Wicko Lake)^7^. Samples were taken from the surface layer to 0.5 m depth and collected seasonally (spring, summer, autumn) in the period 2014–15 (10 lakes) and 2018–19 (3 lakes: Dołgie Małe, Karaś, and Mikoszewskie Lakes). Altogether, 419 lake water samples were collected: Liwia Łuża Lake (30), Resko Lake (30), Jamno Lake (66), Kopań Lake (30), Wicko Lake (48), Gardno Lake (30), Dołgie Małe Lake (6), Dołgie Wielkie Lake (30), Łebsko Lake (66), Sarbsko Lake (30), Ptasi Raj Lake (24), Karaś Lake (12), and Mikoszewskie Lake (17). Samples were taken depending on accessibility and the size of the lake. Precise sampling locations were established according to the longest vertical and horizontal transects conducted across the lake. Central locations were established at the intersection place of transects, which were usually shaped as a single or double cross (i.e.,† or ‡), (Fig. 2).

During the field study, *in-situ* measurements comprised physicochemical parameters of water, including temperature (°C), electrolytic conductivity (EC, µS/cm), salinity (PSU) and total dissolved solids (TDS, mg/L), pH, dissolved oxygen (mg L^−1^ and %) using a pre-calibrated AP-7000 Aquaprobe (AQUARead, UK) multi-parameter sensor. In addition, Secchi disk visibility was employed to assess water transparency (cm).

### Nutrients, Chl-a, and carbon forms

The group of analyzed nutrients consisted of oxidized and reduced forms of nitrogen (N-NO_3_^−^, N-NO_2_^−^, N-NH_4_^+^), as well as oxi (P-PO_4_^3−^, TP). Before the determination of nitrogen forms using an 881 Compact IC Pro (Metrohm, Switzerland) ion chromatograph according to the procedure described in *PN-EN ISO 10304-1:2009*^31^, water samples were filtered through a 0.2-µm sterile syringe filter. The aforesaid nitrogen forms were determined using Metrosep C4 250/4.0 and Metrosep A Supp 250/4.0 analytical columns equipped with, respectively, Metrosep C4 Guard/4.0 and Metrosep A Supp 4/5 Guard pre-columns. Analytical signals were acquired using a conductivity detector. Total phosphorus (TP) and phosphate phosphorus (P-PO_4_^3−^) were determined with ammonium molybdate using a 5001 spectrophotometer (Hitachi, Japan) according to a standard analytical procedure^32^. Total organic carbon (TOC) concentrations were measured in unfiltered samples. Dissolved organic carbon (DOC) was quantified after passing the samples through membrane, nitrocellulose filters with a pore size of 0.45 μm (Millipore). Further analysis was performed after they had been roasted at high temperatures (Shimadzu TOC 5000 analyzer, Japan) according to the protocol described by^33^. Water samples for chlorophyll-a analyses were kept in the dark until analysis (~4 h) to obtain optimal fluorescence intensity. In the laboratory, water was poured into a 25-ml glass cuvette and analyzed using an AlgaeLabAnalyse (ALA) (BBE, Germany) spectral fluorimeter according to the procedure described by^34^.

### Cations and anions

Basic cations (Li^+^, Na^+^, K^+^, Ca^2+^, Mg^2+^) and anions (Br^−^, Cl^−^ and SO_4_^2−^) were determined by ion chromatography according to a standard procedure described in *PN-EN ISO 10304-1:2009*^31^. For the determination of anions, a solution of sodium carbonate and sodium bicarbonate as eluent was used, whereas, for the determination of cations, adipicolinic acid solution was used. The ion chromatography system described above was equipped with an IC 818 pump and IC 863 compact autosampler and run using Magic Net 2.1 software. In the case of basic anions, cations, and nitrogen forms, determination quality was controlled on a daily basis through analysis of the Certified Multielement Ion Chromatography Anion Standard Solution (Fluka Analytical, Switzerland) of lot BCBB8958. For the multielemental standard, the mean errors for the selected ions were 2.2% and 5.6% for accuracy and precision, respectively. To check the accuracy and precision of the chromatographic system, CRMs from environmental samples (HAMIL 20.2 [harbor water] and SUPER-5 [seawater]) were occasionally tested. For the CRMs, the mean error for selected ions was 3.7% and 6.4% for accuracy and precision, respectively. No attempt was made to identify and quantify all ions in the samples (i.e., bicarbonate ions were not measured); therefore it cannot be expected that complete ion balances can be determined. However, ~73% of the results obtained met the criteria for ion balance according to the PN-C-04638-02 standard.

The concentrations of SO_4_^2−^, Br^−^, Cl^−,^ and Na^+^ in the study lakes were stable, without the presence of deviating points. In the case of K^+^ concentration in the lake waters, an outlier result was recorded in only one case. Much more varied results were found for the water concentration of Mg^2+^ ions, which had a large group of outlier results, and of Ca^2+^ and Li^+^ ions, for which a series of extreme points was also recorded.

### Land cover structure of the studied areas

CORINE Land Cover (CLC) data for the catchment areas of the studied lakes were obtained from the CLC 2018 vector layer available in the open database of the CLC project^35^. In Poland, the CLC 2018 project was carried out by the Institute of Geodesy and Cartography and funded by the European Union. The project results were obtained from the website of the General Inspectorate for Environmental Protection (http:\\www.clc.gios.gov.pl). The resources available in the *aPGW* (*Water Management Plan Update*) and *MPHP* (*Map of the Hydrographic Division of Poland*) databases were used to obtain catchment and lake shapes^36,37^. Using the GIS environment (QGIS Desktop 3.24.0 software), vector layers containing objects (polygons) reflecting the extent of each land-cover type were obtained. There were created by trimming the CLC vector layer to the boundaries of each catchment area. The area of objects located within the catchment divide was then recalculated using the attribute table field calculator. Using the data from the attribute tables (area of objects in m^2^ and land-cover-type code from the CLC legend), the area of each land-cover type in m^2^, and their percentage of the total catchment area were recalculated in an Excel spreadsheet. The database contains the percentage of land-cover types at level 1 according to the CLC database legend^35^ in the lake catchment area. Catchment types are also distinguished based on the dominant land-cover type.

The main catchment type of the studied coastal lakes is dominated (in the range 59–78% of the catchment area) by agricultural areas, followed by the semi-natural or forest type. Four of the study lakes have catchments of mixed agricultural and semi-natural land-cover types (39–51%). The highest proportion of heavily transformed components in the catchment type is found in the Jamno Lake catchment, where they account for as much as 10% of the area (for the other lakes, this proportion is in the 0–5% range). Water bodies occupy a maximum of 23% of the catchment area, and the proportion of wetlands in the catchments of the studied lakes is negligible (0–8%). Among the studied lakes, only Dołgie Małe has only a direct catchment.

## Data Records

The data collected were published in an open-access repository^38^. Four files were included in the repository: *BCLs_database.xlsx*, *BCLs_meteo.xlsx*, *BCLs_physchem_CLC.gpkg* and *Description_of_attribute_table.xlsx*. The file *BCLs_database.xlsx* is a database for the lakes described in this paper and contains: lake name; month and year of sampling; total lake catchment area; percentage of each land-cover type in the catchment; catchment type according to dominant land-cover type; lake area, size class, and hydrological connectivity; existing forms of protection; sample number; longitude and latitude; lake zone from which the sample was taken; and all physicochemical parameters mentioned in this article (Supplementary Table 1). *BCLs_physchem_CLC.gpkg* is a geo-packed file that can be opened in a GIS environment. It contains lake outlines (vector layer), total catchment outlines (vector layer), raster layers containing land cover (CLC) clipped to the surface of each catchment, and a multipoint layer containing the plotted points of each sampling event with attributes relating to the measurement values of all analyzed parameters (identical to the information contained in *BCLs_database.xlsx*). This file additionally contains a multipoint layer with meteorological data for each water sampling date (air temperature, cloud cover, air pressure, and wind direction and strength) from weather stations along the Baltic Sea coast (weather stations at Gdańsk-Świbno, Łeba, Darłowo, and Kołobrzeg) provided by the Institute of Meteorology and Water Management (IMGW-PIB, Poland). They supplemented the information on the meteorological situation prevailing on the lakes in the sampling period. However, they provide only a background for understanding the physicochemical parameter values obtained for the waters and an indication of the complex conditions of water exchange with the sea. The same meteorological data have been included in an additional excel file *BCLs_meteo.xlsx*. Each of the layers used in *BCLs_physchem_CLC.gpkg* has metadata described along with information about the authors/source of each layer. *Description_of_attribute_table*.xlsx provides an explanation of the abbreviated variable names in the attribute table headings and a description of the variables.

## Technical Validation

All analyses were carried out in accordance with the standards (ISO, APHA) listed in the Methods or the procedures specified in the scientific publications cited, and certified standard solutions were used in the case of ion chromatography analysis. To ensure the quality of measurements, all instruments were calibrated according to producers’ recommendations.

**Fig. 3:**
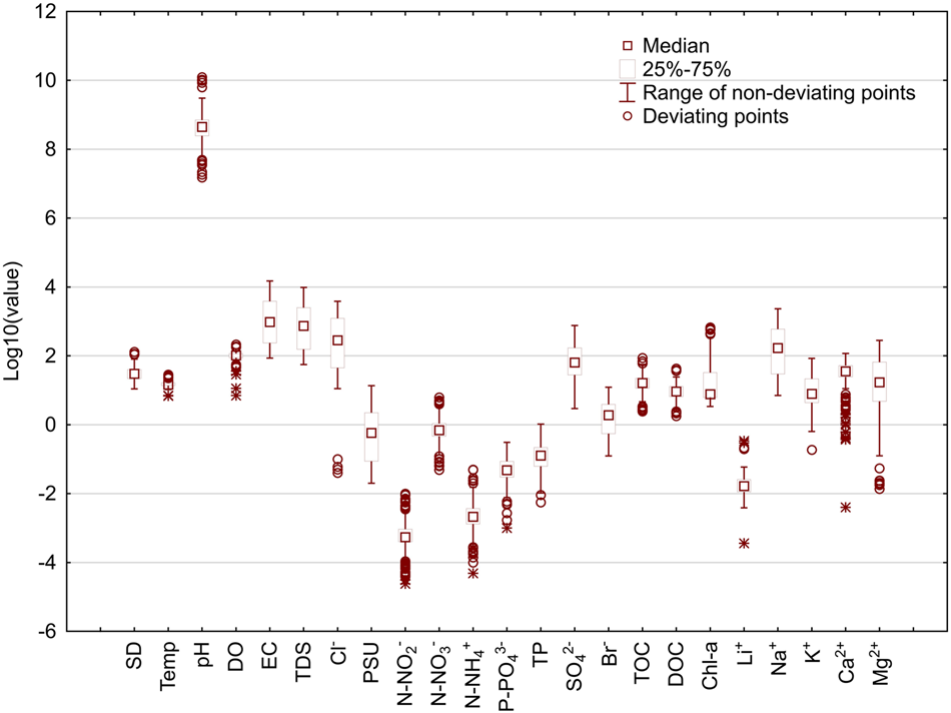
Distribution of results of measurements of physicochemical parameters (presented on a logarithmic scale). Box-and-whisker plot shows distribution of analysis results for all physicochemical parameters of sampled water.

To present descriptive statistics (mean, median, minimum, maximum, upper and lower quartile, standard deviation) for all the physiochemical parameters included in the database, a table was prepared (Table 1). A box-and-whisker plot on a logarithmic scale was used to visualize the distribution of the measurement results obtained for each physicochemical parameter, together with the determination of deviating points and extreme values (Fig. 3). The statistical software Statistica v. 13.3 (TIBCO, Palo Alto, CA, USA) was used for this purpose.

**Table 1:** Descriptive statistics of presented data: mean, median, range, lower quartile, upper quartile, units.

Variables	Units	N	Data with values (%)	Mean	Median	Minimum	Maximum	Lower quartile	Upper quartile	Standard deviation
Secchi depth	cm	419	100.0	34	30	11	130	22	40	19
Water temp	°C	419	100.0	16.4	14.7	6.9	28.4	13.7	16.5	4.3
pH	—	419	100.0	8.62	8.65	7.18	10.09	8.39	8.85	0.43
DO	%	419	100.0	103.02	103.31	7.04	212.90	88.30	116.30	23.81
EC	µS/cm	419	100.0	2628.08	960.00	86.36	14934.20	236.33	3824.68	3409.62
TDS	mg L^−1^	419	100.0	1720.52	740.00	56.00	9707.00	153.86	2498.50	2203.51
Cl^-^	mg L^−1^	419	100.0	753.70	281.74	0.04	3822.89	45.05	1229.07	927.56
Salinity	PSU	419	100.0	1.492	0.578	0.020	13.674	0.087	2.236	2.140
N-NO_2_^−^	mg L^−1^	419	100.0	0.00097	0.00054	0.00002	0.00999	0.00040	0.00094	0.00146
N-NO_3_^−^	mg L^−1^	419	100.0	0.930	0.693	0.049	6.229	0.463	1.081	0.830
N-NH_4_^+^	mg L^−1^	419	100.0	0.003	0.002	0.000	0.049	0.001	0.004	0.005
P-PO_4_^3−^	mg L^−1^	419	100.0	0.068	0.047	0.000	0.310	0.029	0.086	0.059
TP	mg L^−1^	419	100.0	0.160	0.127	0.006	1.044	0.062	0.219	0.133
SO_4_^2−^	mg L^−1^	419	100.0	119.96	64.00	2.93	765.98	27.81	170.89	135.08
Br^−^	mg L^−1^	419	100.0	2.68	1.90	0.13	12.30	0.55	3.90	2.58
TOC	mg L^−1^	419	100.0	18.81	16.34	2.44	86.90	11.56	22.21	11.73
DOC	mg L^−1^	419	100.0	10.47	9.25	1.78	42.93	6.60	12.81	5.45
Chl-a	mg L^−1^	419	100.0	37.56	7.66	3.41	670.66	6.20	32.47	80.04
Li^+^	mg L^−1^	419	100.0	0.022	0.017	0.000	0.338	0.011	0.025	0.027
Na^+^	mg L^−1^	419	100.0	397.59	168.64	7.03	2330.69	29.73	598.70	515.26
K^+^	mg L^−1^	419	100.0	16.02	7.87	0.19	84.38	4.40	21.64	17.03
Ca^2+^	mg L^−1^	419	100.0	39.53	35.40	0.00	117.32	24.58	51.87	22.56
Mg^2+^	mg L^−1^	419	100.0	44.56	17.00	0.01	279.63	4.81	65.66	55.52

### Supplementary information

Supplementary Table 1

## Data Availability

No custom code was generated for the purposes of our paper.
